# Dual DNA Barcoding for the Molecular Identification of the Agents of Invasive Fungal Infections

**DOI:** 10.3389/fmicb.2019.01647

**Published:** 2019-07-18

**Authors:** Minh Thuy Vi Hoang, Laszlo Irinyi, Sharon C. A. Chen, Tania C. Sorrell, The ISHAM Barcoding of Medical Fungi Working Group, Michael Arabatzis, Wieland Meyer

**Affiliations:** National and Kapodistrian University of Athens, Athens, Greece; PathWest Laboratory Medicine, Nedlands, WA, Australia; Universitat Rovira i Virgili, Reus, Spain; Universitàdegli Studi di Perugia, Perugia, Italy; Universidad Nacional Autónoma de México, Mexico City, Mexico; Westmead Hospital, University of Sydney, Sydney, NSW, Australia; Agriculture and Agri-Food Canada, Ottawa, ON, Canada; Chulalongkorn University, Bangkok, Thailand; Universidade Federal de São Paulo, São Paulo, Brazil; Institut Pasteur, Paris, France; FABI, Pretoria, South Africa; Westerdijk Fungal Biodiversity Institute, Utrecht, Netherlands; Institut Pasteur, Paris, France; Institut Pasteur, Paris, France; University of the Free State, Bloemfontein, South Africa; Universitat Rovira i Virgili, Reus, Spain; University of Sydney/Westmead Hospital, Sydney, NSW, Australia; BCCM/IHEM, Scientific Institute of Public Health, Brussels, Belgium; The Field Museum, Chicago, IL, United States; Westmead Hospital, University of Sydney, Sydney, NSW, Australia; Agriculture and Agri-Food Canada, Ottawa, ON, Canada; Westmead Hospital, University of Sydney, Sydney, NSW, Australia; University of Campinas, Campinas, Brazil; Instituto de Pesquisa Clínica Evandro Chagas (IPEC), Fundação Oswaldo Cruz (FIOCRUZ), Rio de Janeiro, Brazil; Universidade Federal de São Paulo, São Paulo, Brazil; Universidade Federal de São Paulo, São Paulo, Brazil; Aix-Marseille University, Marseille, France; University of Minho, Braga, Portugal; Aix-Marseille University, Marseille, France; Aix-Marseille University, Marseille, France; National Center for Biotechnology Information, Bethesda, MD, United States; Westerdijk Fungal Biodiversity Institute, Utrecht, Netherlands; National Center for Biotechnology Information, Bethesda, MD, United States; Agriculture and Agri-Food Canada, Ottawa, ON, Canada; Universidade Federal de Goiás, Goiânia, Brazil; Westmead Hospital, University of Sydney, Sydney, NSW, Australia; National Center for Biotechnology Information, Bethesda, MD, United States; BCCM/IHEM, Scientific Institute of Public Health, Brussels, Belgium; Universidad Nacional Autónoma de México, Mexico City, Mexico; Universidad Nacional Autónoma de México, Mexico City, Mexico; National and Kapodistrian University of Athens, Athens, Greece; Kasetsart University, Bangkok, Thailand; Instituto de Pesquisa Clínica Evandro Chagas (IPEC), Fundação Oswaldo Cruz, FIOCRUZ, Rio de Janeiro, Brazil.; ^1^Molecular Mycology Research Laboratory, Centre for Infectious Diseases and Microbiology, Sydney Medical School, Westmead Clinical School, Faculty of Medicine and Health, The University of Sydney, Sydney, NSW, Australia; ^2^The Westmead Institute for Medical Research, Westmead, NSW, Australia; ^3^Marie Bashir Institute for Infectious Diseases and Biosecurity, The University of Sydney, Sydney, NSW, Australia; ^4^Centre for Infectious Diseases and Microbiology Laboratory Services, Institute for Clinical Pathology and Medical Research, NSW Health Pathology, Westmead, NSW, Australia; ^5^Research and Education Network, Westmead Hospital, Westmead, NSW, Australia

**Keywords:** identification, fungal DNA barcoding, dual barcoding system, internal transcribed spacer region, *translational elongation factor 1α*, ISHAM Barcoding Database, invasive fungal diseases

## Abstract

Invasive fungal infections, such as aspergillosis, candidiasis, and cryptococcosis, have significantly increased among immunocompromised people. To tackle these infections the first and most decisive step is the accurate identification of the causal pathogen. Routine identification of invasive fungal infections has progressed away from culture-dependent methods toward molecular techniques, including DNA barcoding, a highly efficient and widely used diagnostic technique. Fungal DNA barcoding previously relied on a single barcoding region, the internal transcribed spacer (ITS) region. However, this allowed only for 75% of all fungi to be correctly identified. As such, the *translational elongation factor 1α* (*TEF1α*) was recently introduced as the secondary barcode region to close the gap. Both loci together form the dual fungal DNA barcoding scheme. As a result, the ISHAM Barcoding Database has been expanded to include sequences for both barcoding regions to enable practical implementation of the dual barcoding scheme into clinical practice. The present study investigates the impact of the secondary barcode on the identification of clinically important fungal taxa, that have been demonstrated to cause severe invasive disease. Analysis of the barcoding regions was performed using barcoding gap analysis based on the genetic distances generated with the Kimura 2-parameter model. The secondary barcode demonstrated an improvement in identification for all taxa that were unidentifiable with the primary barcode, and when combined with the primary barcode ensured accurate identification for all taxa analyzed, making DNA barcoding an important, efficient and reliable addition to the diagnostic toolset of invasive fungal infections.

## Introduction

While AIDS-associated *Pneumocystis jirovecii* pneumonia (Pjp) and cryptococcosis have declined in developed countries due to widespread use of highly active antiretroviral treatment (Dromer et al., 2004; Morris et al., 2004; Rajasingham et al., 2017), the overall burden of invasive fungal diseases (IFDs), especially candidemia and invasive aspergillosis has increased worldwide (Patterson, 2005; Maschmeyer, 2006; Pfaller et al., 2006b; Warnock, 2007; Pappas et al., 2010; Benedict et al., 2017). IFDs alone cause about 1.6 million deaths/year (Brown et al., 2012). The rise in incidence of IFDs is largely due to an increase in at-risk populations, especially immunocompromised individuals, such as recipients of solid organs or hematopoietic stem cell transplants, and patients with underlying chronic diseases (Kontoyiannis et al., 2010; Brown et al., 2012; Armstrong-James et al., 2014; Bitar et al., 2014; Schelenz et al., 2015). It is paramount that the management of invasive mycoses must be improved through advancements in prevention, diagnosis, treatment and surveillance (Denning, 2016; Kneale et al., 2016; Cole et al., 2017).

The majority of the current fungal diagnostic techniques are inadequate for the identification of all pathogenic fungi, which is a pre-requisite for timely initiation of appropriate antifungal therapy (Schelenz et al., 2015; Irinyi et al., 2016; Cole et al., 2017). Culture-based identification techniques rely on morphological and phenotypic characteristics, are often inaccurate, and lack in most cases species-specific features. Phenotypic traits also fail to differentiate between closely related species or species complexes with near-identical morphological characteristics but distinguishable genetic traits, such as the casual agents of many mold infections, including aspergillosis and scedosporiosis (Tavanti et al., 2005). Further, not all pathogenic fungi grow under laboratory conditions. Morphology based diagnostics are also time-consuming (7–14 days), highly laborious, and heavily dependent on the level of mycological expertise of the microscopist, making them unsuitable for rapid and reliable diagnosis (Irinyi et al., 2016).

Serological tests or fungal biomarkers, such as the *Aspergillus* antigen test or the *Cryptococcus* lateral flow assay (CrAg^®^ LFA, IMMY, Norman, OK, United States), are available, but they are designed to identify specific pathogens (Chen et al., 2014; Schelenz et al., 2015; Cadena et al., 2016).

To overcome the limitations of standard phenotypic diagnosis and identification methods, culture- and “expert-free” methods capable of identifying fungi directly from biological specimens are needed. Sequence-based identification has proven to be more accurate than conventional methods in diagnostic clinical mycology (Ciardo et al., 2006; Balajee et al., 2007). Among the applied molecular techniques, DNA barcoding is one of the most promising and efficient methods, as it enables rapid identification of species and recognition of cryptic species across all fungal genera. As such, DNA barcoding has recently been established as the gold standard identification technique for fungal species and has been proven to be more accurate than conventional identification techniques (Ciardo et al., 2006; Balajee et al., 2007). Barcodes are standardized, easily amplified, universal short DNA sequences (500–800 bp), which are divergent at the species level enabling rapid identification by comparison with a validated reference sequence collection. To ensure consistency of identification, barcodes should be unique to a single species, and stable within each species (Hebert et al., 2003). Additionally, interspecies variation must exceed the intraspecies variation, generating a “break” in the distribution of distances, which is referred to as the “barcoding gap” (Meyer and Paulay, 2005).

**FIGURE 1:**
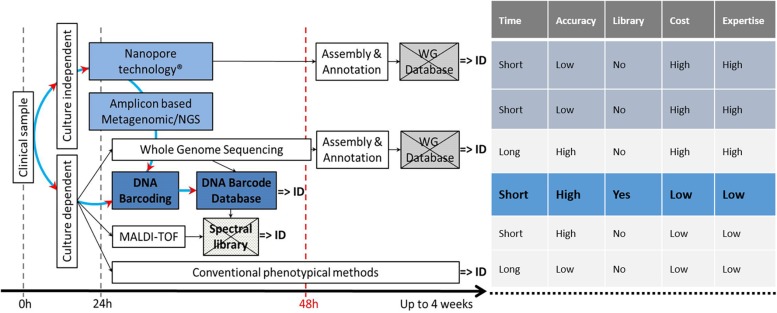
Current and potentially available techniques for fungal ID, showing the central role of the reference DNA barcode database and the pros and cons of all techniques. Crossed boxes indicate missing databases. DNA barcoded reference strains are fundamental to build up a spectral library.

DNA barcoding and its associated references databases [BOLD (Hebert et al., 2003), UNITE (Kõljalg et al., 2013), RefSeq at Genbank (Schoch et al., 2014), and the “ISHAM Barcoding Database” (Meyer et al., 2018)], plays a central role in the identification landscape, as it is the basis for all future methods, either culture dependent or culture independent (Figure 1).

**FIGURE 2:**
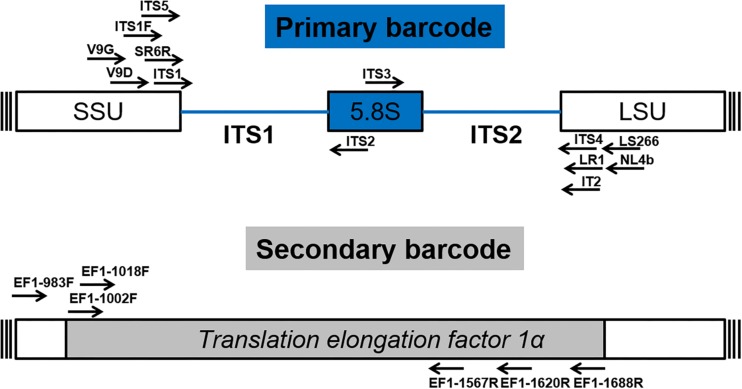
Schematic structure of the primary (ITS) and secondary (*TEF1α*) fungal DNA barcode regions indicating universal primers for their amplification.

After numerous candidate genetic loci were evaluated, with varying success rates, the internal transcribed spacer (ITS) region was established as the primary fungal DNA barcode by Schoch et al. (2012). The ITS region is composed of two non-coding and variable regions, ITS1 and ITS2, flanking the highly conserved 5.8S gene. They are located between the 18S [small subunit (SSU)] and 28S [large subunit (LSU)] genes in the nrDNA repeat (White et al., 1990). The advantage of the ITS region is, that it can be easily amplified from most fungal taxa, using universal primers, with the most commonly used ones being the ITS1, ITS2, ITS3, ITS4, and ITS5 (White et al., 1990). Fungal-specific primers were also designed to avoid cross reactivity with plant or animal DNA, such as SR6R and LR1 (Vilgalys and Hester, 1990), V9D, V9G, and LS266 (Gerrits van den Ende and de Hoog, 1999), IT2 (Beguin et al., 2012), ITS1F (Gardes and Bruns, 1993) and NL4b (O’Donnell, 1993; Figure 2 and Table 1). To provide quality controlled reference primary fungal DNA barcode sequences the International Society for Human and Animal Mycology (ISHAM) “ITS DNA barcode database” was established in 2015 (Irinyi et al., 2015). The primary fungal DNA barcode region identifies up to 75% of the estimated ∼700 pathogenic fungal species (de Hoog et al., 2014).

**TABLE 1:** Universal and fungal specific primers for the dual DNAbarcoding scheme.

**Barcode**	**Primer name**	**Sequence**	**References**
Primary barcode	ITS1	5′ TCCGTAGGTGAACCTGCGG 3′	White et al.,1990
	ITS2	5′ GCTGCGTTCTTCATCGATGC 3′	White et al.,1990
	ITS3	5′ GCATCGATGAAGAACGCAGC 3′	White et al.,1990
	ITS4	5′ TCCTCCGCTTATTGATATGC 3′	White et al.,1990
	ITS5	5′ GGAAGTAAAAGTCGTAACAAGG 3′	White et al.,1990
	ITS1F	5′ CTTGGTCATTTAGAGGAAGTAA 3′	Gardes and Bruns,1993
	IT2	5′ CCTCCGCTTATTGATATGCTTAGG 3′	Beguin et al.,2012
	SR6R	5′ AAGTATAAGTCGTAACAAGG 3′	Vilgalys and Hester,1990
	LR1	5′ GGTTGGTTTCTTTCCT 3′	Vilgalys and Hester,1990
	V9D	5′ TTAAGTCCCTGCCCTTTGTA 3′	Gerrits van den Ende and de Hoog,1999
	V9G	5′ TACGTCCCTGCCCTTTGTA 3′	Gerrits van den Ende and de Hoog,1999
	LS266	5′ GCATTCCCAAACAACTCGACTC 3′	Gerrits van den Ende and de Hoog,1999
	NL4b	5′ GGATTCTCACCCTCTATGAC 3′	O’Donnell,1993
Secondary barcode	EF1-1002F	5′ TTCATCAAGAACATGAT 3′	Stielow et al.,2015
	EF1-1018F	5′ GAYTTCATCAAGAACATGAT 3′	Stielow et al.,2015
	EF1-1620R	5′ GACGTTGAADCCRACRTTGTC 3′	Stielow et al.,2015
	EF1-1688R	5′ GCTATCATCACAATGGACGTTCTTGGAG 3′	Stielow et al.,2015
	EF1-983F	5′ GCYCCYGGHCAYCGTGAYTTYAT 3′	Rehner and Buckley,2005
	EF1-1567R	5′ ACHGTRCCRATACCACCRATCTT 3′	Rehner and Buckley,2005

To address the shortcomings of the ITS region and hence close this identification gap, a secondary barcode was proposed in 2015 (Stielow et al., 2015). The *translational elongation factor 1α* (*TEF1α*) was selected due to its high species discrimination across fungal taxa and the ability to design universal primers, such as EF1-1018F (Al33F)/EF1-1620R (Al33R), EF1-1002F (Al34F)/EF1-1688R (Al34R) (Stielow et al., 2015), or EF1-983F/EF1-1567R (Rehner and Buckley, 2005; Figure 2 and Table 1). To accommodate the secondary fungal DNA barcode a dedicated database that would eventually include all medically relevant fungal species was established to complement the ISHAM-ITS database, which contains only quality-controlled *TEF1α* sequences obtained from taxonomically verified fungal cultures (Meyer et al., 2018). Both databases have been combined within the “ISHAM Barcoding Database”,^1^ which was launched in November 2017 at the 7th International DNA barcoding conference at the Kruger National Park in South Africa. The combined dual barcode database provides the medical and veterinary community with quality-controlled primary and secondary fungal DNA barcodes reference sequences. This database is publicly available, and its quality-controlled sequences are shared with other major databases, including RefSeq within GenBank (Benson et al., 2014) and UNITE (Kõljalg et al., 2013).

To date, the clinical utility and accuracy of the dual DNA barcoding system for identification of pathogenic fungi has not been assessed. This study aimed to increase the number of reference secondary barcode sequences for pathogenic fungal species and to compare the accuracy and resolution of the primary and secondary fungal DNA barcodes separately or in combination, focusing on fungi causing invasive fungal infections.

## Materials and Methods

### Cultures

To generate quality controlled *TEF1α* sequences 270 strains, representing 90 human/animal pathogenic fungal species, were used (see Supplementary Table 1).

### DNA Extraction

DNA was isolated and purified from cultures using either previously described manual method (Ferrer et al., 2001) or the Quick-DNA Fungal/Bacterial Kit (D6007, Zymo Research) according to the manufacturer’s instructions.

### DNA Barcode Generation

The secondary fungal DNA barcoding region (*TEF1α*) was amplified using the primers described by Stielow et al. (2015), including: EF1-1018F (Al33F) (5′ GAYTTCATCAAGAACATGAT 3′) and EF1-1620R (Al33R) (5′ GACGTTGAADCCRACRTTGTC 3′) being used together and EF1-1002F (Al34F) (5′ TTC ATCAAGAACATGAT 3′) and EF1-1688R (Al34R) (5′ CTATCATCACAATGG ACGTTCTTGGAG 3′) being used together (Stielow et al., 2015). The primer set Al33F-Al33R was first used to amplify the *TEF1α* region. If amplification was unsuccessful then it was repeated using the second primer set Al34F-Al34R. Both primer pairs used the following PCR amplification protocol: 5 min initial denaturing at 94°C, followed by 40 of 50 s at 94°C, 50 s annealing at 48°C, 50 s at 72°C and 7 min final extension at 72°C (Stielow et al., 2015). Amplification success was visualized through gel electrophoresis in a 1.5% agarose gel containing ethidium bromide (EtBr) with ultra-violet illumination. Successfully amplified PCR products were sent for commercial sequencing, e.g., Macrogen Inc., South Korea in both forward and reverse directions. Bidirectional sequenced were assembled and edited using Sequencher^®^ ver. 5.3. (Gene Codes Corporation, Ann Arbor, MI, United States). Sequences were manually checked to resolve ambiguous bases on the forward and reverse trace files considering the PHRED scores received.

### DNA Barcode Analysis

The sequences for each taxon were aligned with the program CLUSTALW (Thompson et al., 1994) part of the software MEGA ver. 7 (Larkin et al., 2007). Resulting alignments were checked visually and edited when needed.

The intraspecies diversity was estimated by calculating the average nucleotide diversity (π) within species, where there were sequences available from more than three strains. The proportion of nucleotide differences in all haplotypes in the sample was derived using the software DnaSP ver. 5.10.01 (Librado and Rozas, 2009).

Individual analysis of the primary and secondary barcoding regions was performed through barcoding gap analysis. Additionally, analysis was performed on the combined barcoding regions. Taxonomic groups were selected for analysis if represented by three or more species, and if those species were represented by three or more strains. Furthermore, taxonomic groups were required to include at least one species that has been demonstrated to cause invasive fungal infections. Only strains with both primary and secondary barcodes in the database where included in the analysis. When available, more specific taxonomic groups such as clades were selected over genera. Sequences for each genus were aligned and cut to equal length using CLUSTALW as present in MEGA ver. 7 (Larkin et al., 2007; Kumar et al., 2016). Genetic distances between each strain were calculated using the Kimura 2-parameter model (K2P) and the intraspecies and interspecies genetic distances were compared (Kimura, 1980). Intra- and interspecies genetic distances were graphed against frequency and analyzed for barcoding gaps. Barcoding gaps were defined by the presence of a distinct difference between the largest intraspecies genetic distance and smallest interspecies genetic distance (Irinyi et al., 2015), i.e., there was no common x value between the intra- and interspecies groups.

The introduction of the secondary fungal DNA barcode was accessed for each genus on the basis that there would be an improvement in DNA barcoding if the secondary barcode generated a barcoding gap when the primary barcode did not, or if the overlap between the intra- and interspecies genetic distances was reduced.

## Results

The study produced 270 new quality controlled secondary fungal barcode sequences, covering 90 pathogenic fungal species (Supplementary Table 1). Overall, the PCR success rate was high within the 270 *TEF1* sequences. 220 secondary barcodes were generated using the Al33F–Al33R primer pair and 50 were amplified with the Al34F–Al34R primer pair. There was no trend in amplification success in different fungal species. However, the Al34F–Al34R primer set was required to amplify all strains of *Aspergillus niger*, *Candida albicans*, *Candida dubliniensis*, *Kluyveromyes marxianus*, and *Pichia kudriavzevii*. There was unsuccessful amplification with both primer sets for some strains of *Cladosporium* spp., *Rhodotorula* spp., and *Trichosporon* spp.

All sequences were submitted to the ISHAM Barcoding Database (see footnote 1). The length of the ITS and partial *TEF1α* sequences in the database ranges 285–791 and 534–1002 bp, respectively.

**FIGURE 3:**
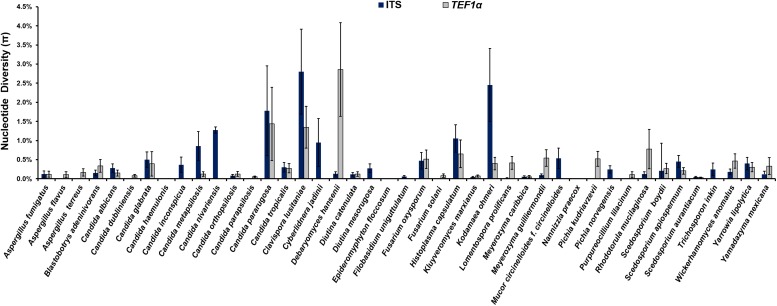
Intraspecies variation for species, which are represented by more than three strains, in the internal transcribed spacer (ITS) (blue bars) primary fungal DNA barcode compared with the *translation elongation factor 1α* (*TEF1α*) (gray bars) secondary fungal DNA barcode.

The analysis of the nucleotide diversity (π) of 43 fungal species with more than three strains in the ISHAM barcoding database showed that the *TEF1α* region is less diverse than the ITS region in most species (Figure 3). The intraspecies variation of *TEF1α* was for most species below 1.5%, confirming the secondary barcode as a more discriminator marker. According to the selection criteria four different taxonomic groups were selected as proof of principle for the dual barcoding system. These included the two genera, *Diutina* and *Scedosporium*, and the two taxonomic clades, *Lodderomyces* and *Pichia*. The *Diutina* genus was selected for analysis despite one of the species, *Diutina rugosa*, being represented by only two strains as this genus is newly established that causes rare disease (Table 2). The species and number of strains included in these analyses are outlined in Table 2.

**TABLE 2:** Number of fungal strains with primary and secondarybarcodes present in the ISHAM Barcoding Databaseanalyzed in this study.

**Taxonomic group**	**Species/Species complex**	**Number of strains**
*Diutina*	*Diutina catenulata*	11
	*Diutina mesorugosa*	9
	*Diutina rugosa*	2
*Lodderomyces* clade	*Candida albicans*	13
	*Candida dubliniensis*	12
	*Candida metapsilosis*	7
	*Candida orthopsilosis*	11
	*Candida parapsilosis*	26
	*Candida tropicalis*	16
*Pichia* clade	*Candida inconspicua*	4
	*Pichia kudriavzevii*	15
	*Pichia norvegensis*	10
*Scedosporium*	*Scedosporium apiospermum*	6
	*Scedosporium aurantiacum*	13
	*Scedosporium boydii*	4

### *Diutina* Species

**FIGURE 4:**
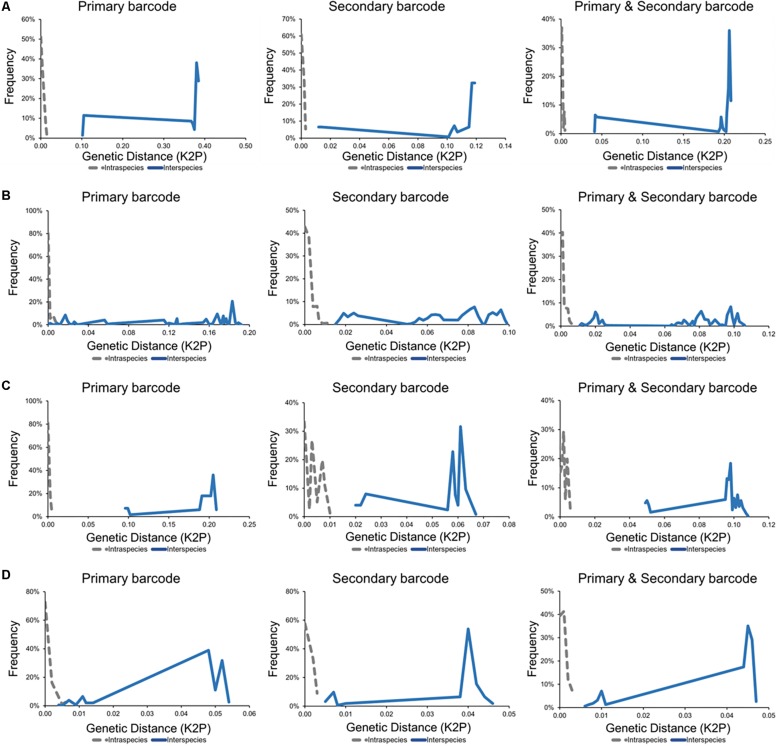
Barcoding gap analyses for fungal taxa including causative agents of invasive fungal disease with intraspecies (gray) and interspecies (blue) genetic distances calculated using the Kimura 2-parameter model. **(A)** *Diutina* genus (*Diutina catenulata, Diutina mesorugosa*, and *Diutina rugosa*). **(B)** *Lodderomyces-*clade (*Candida albicans*, *Candida dublinsiensis, Candida metapsilosis, Candida orthopsilosis, Candida parapsilosis*, and *Candida tropicalis*). **(C)** *Pichia-*clade (*Candida inconspicua, Pichia kudriavzevii*, and *Pichia norvegensis*). **(D)** *Scedosporium* genus (*Scedosporium apiospermum, Scedosporium aurantiacum*, and *Scedosporium boydii*).

Barcoding gap analysis of the genus *Diutina* demonstrated no overlap between the intraspecies and interspecies genetic distances with either the primary or secondary fungal DNA barcodes (Figure 4). There was similarly no overlapping region for the barcoding gap analysis of the combination of the primary and secondary barcodes (Figure 4). As such, both fungal barcodes and the combination of the barcodes generated appropriate barcoding gaps.

### The *Lodderomyces* Clade

For the *Lodderomyces* clade, the intraspecies and interspecies genetic distances, generated by the primary fungal barcode produced an overlapping region thereby indicating that there was no barcoding gap (Figure 4). The barcoding gap analysis for the secondary DNA barcode (*TEF1α*) demonstrated no overlap in the intraspecies and interspecies genetic distances for *Lodderomyces* thus showing a clear barcoding gap (Figure 4). The *Lodderomyces* clade analysis using the combination of both the ITS and *TEF1α* regions resulted in the generation of a barcoding gap being generated (Figure 4).

### The *Pichia* Clade

Barcoding gap analysis of the *Pichia* clade did not reveal any overlapping regions in the genetic distances for either the ITS or *TEF1α* regions (Figure 4). As there were no overlapping regions this demonstrated that there are barcoding gaps for both the primary and secondary barcode. These results were similar when both barcoding regions were combined as there was a barcoding gap produced (Figure 4).

### *Scedosporium* Species

Barcoding gap analysis of the primary fungal DNA barcode showed an overlapping region (Figure 4). For the secondary barcode, the barcoding gap analysis revealed that there was no overlap between the intraspecies and interspecies genetic distances and so a barcoding gap was present (Figure 4). The barcoding gap analysis of the combination of both barcodes, produced a barcoding gap as there was no overlap between the intraspecies and interspecies genetic distances (Figure 4).

## Discussion

### ISHAM Barcoding Database Expansion

Accurate and rapid routine diagnostic tools are essential to reduce the burden of fungal disease. DNA barcoding requires reliable quality-controlled reference sequences for its implementation. As such, fungal DNA Barcoding databases need to have high taxonomic coverage and reliable sequences to ensure users can accurately match sample sequences to the correct reference sequences. Additionally, the more sequences from different strains for each species are represented, the higher is the accuracy of the identification, as this reflects more realistically the existing species variation.

In this study, we generated 270 secondary barcodes, which could be amplified reliably using either the primer set Al33F–Al33R or the primer set Al34f–A134R (Figure 2), with a few exceptions where no amplifications where obtained for some strains of *Cladosporium* spp.*, Rhodotorula* spp., or *Trichosporon* spp. In those cases, modification of the amplification conditions, such as touchdown PCR, may be appropriate.

As a result of this study the ISHAM Barcoding Database contains, as of the May 10, 2019, 4091 primary (ITS) and 868 secondary barcode (*TEF1α*) sequences covering 640 and 129 pathogenic fungal species, respectively. From those 2681 ITS and 613 *TEF1α* sequences representing 252 species cause invasive fungal diseases. There are an estimated 700 pathogenic fungal species, with the majority rarely causing infections. As such, the taxonomic coverage of ITS sequences within the “ISHAM Barcoding Database” is close to covering all pathogenic fungal species, whilst the *TEF1α* sequences cover less than one fifth. In addition, 59 species are currently only represented by one *TEF1α* sequence, which limits identification due to underrepresented intraspecies variation. As the “ISHAM Barcoding Database” has only been recently expanded to include *TEF1α* sequences, it is expected that as more *TEF1α* sequences are submitted, these issues will be resolved. Currently the “ISHAM Barcoding Database” has enabled pragmatic implementation of the dual barcode scheme for the identification of pathogenic fungi.

### Comparison of the Primary and Secondary Barcodes

Since the establishment of the secondary fungal DNA barcode the improvement of the identification accuracy for pathogenic fungi has not been assessed. In this study, we compared for the first time the primary and secondary fungal DNA barcodes via barcoding gap analysis based on intraspecies and interspecies genetic distance values as calculated using the K2P model (Kimura, 1980). In addition, the combination of both barcodes was tested and compared to the individual barcodes.

#### *Diutina* Species (Previously Belonging to the Genus *Candida*)

The genus *Diutina* was established only recently. This group of fungal species was previously part of the genus *Candida* (Kurtzman et al., 2011; Khunnamwong et al., 2015). *Diutina* species are uncommon agents of disease, but are associated with nosocomial infections, with fatality rates of up to 70% (Padovan et al., 2013). *Diutina catenulata* and *Diutina rugosa* are well known causes of fungemia in immunocompromised patients and the seriously ill (Radosavljevic et al., 1999; Minces et al., 2009; Behera et al., 2010; Ha et al., 2018). *D. rugosa* is notable for its increasing resistance against multiple antifungal agents worldwide, thereby increasing the need for early detection and identification to enable timely initiation of effective treatment (Pfaller et al., 2006a).

An exception was made for the genus *Diutina* to be included into this analysis, as *D. rugosa* is only represented by two strains in the analysis (Table 2). The inclusion of the species was important as *Diutina mesorugosa* and *D. rugosa* have been proposed as synonyms with the variability of ITS sequences used as supporting evidence (Ming et al., 2019).

Barcoding gap analysis indicates that these species can be clearly identified as separate species. The *Diutina* species are representative of the 75% of pathogenic fungi that are accurately identified by the primary barcode (Irinyi et al., 2016). The secondary barcode and the combination of both barcodes equally identified these species (Figure 4), demonstrating the same level of resolution as the primary barcode (Stielow et al., 2015).

#### The *Lodderomyces* Clade

The *Lodderomyces* clade contains 21 *Candida* species, including some of the major pathogenic fungal species (Kurtzman et al., 2011). This clade contains three of the five most common pathogenic *Candida* species: *Candida albicans*, *Candida dubliniensis*, and *Candida parapsilosis* (Spampinato and Leonardi, 2013). These species cause a wide spectrum of diseases, from cutaneous infections to fatal disseminated septicemia (Tavanti et al., 2005). *Candida* species are predominantly commensals in the body and as such these infections largely target immunocompromised patients and are commonly the cause of nosocomial outbreaks (Brown et al., 2012).

*Candida* is a polyphyletic genus with a highly complex taxonomic history (Kurtzman et al., 2011). In the latest edition of The Yeasts A Taxonomic Study, 314 different *Candida* species were described excluding species with anamorph-teleomorph linkages (Kurtzman et al., 2011). The genus *Candida* was initially intended to include undifferentiated yeasts that cannot be identified by phenotype (Berkhout, 1923). As such there is little stability in the genus with some species being linked to others as anamorphs and the introduction of newly found species. With the advanced use of genomics in taxonomic studies the composition of species and the *Lodderomyces* clade are expected to change.

The barcoding gap analysis of the primary barcode was unable to generate a barcoding gap and so was unable to accurately identify all species of the clade (Figure 4). Upon closer inspection, the lack of a barcoding gap was due to the close relationships between *Candida orthopsilosis* and *Candida parapsilosis*, which form part of the *Candida parapsilosis* species complex. This species complex was previously representing a single species, *Candida parapsilosis*, with three subgroups that were then revised to be separate species based on various molecular techniques, including sequencing of the ITS region. The barcoding gap analysis of this study, however, did not reflect this differentiation and so ITS was unable to identify these different species. Barcoding gap analysis of the secondary barcode did produce a barcoding gap and thereby demonstrated that *TEF1α* could accurately identify all species of the Lodderomyces clade (Figure 4). As such, the introduction of the dual barcoding scheme resulted in an accurate identification of these pathogenic fungal species.

#### The *Pichia* Clade

The *Pichia* clade is composed of 20 species. In our study, *Candida inconspicua* was also included in the analysis, as it is thought to be the anamorph for *Pichia cactophila* (Kurtzman and Robnett, 1998; Kurtzman et al., 2008, 2011). *Pichia kudriavzevii* and *Pichia norvegensis* are predominantly linked to nosocomial pathogens and may cause outbreaks of infections (Nagarathnamma et al., 2017). *Candida inconspicua* has also been found in clinical samples whilst the other species of the *Pichia* clade have not yet been found to be medically relevant (Kurtzman et al., 2011; Guitard et al., 2013). These species also cause invasive infections in patients with a highly compromised immune systems, and are often found under previous names in the literature (Kurtzman et al., 2011; Guitard et al., 2013; Schuster et al., 2013; Sanclemente et al., 2015; Douglass et al., 2018).

Similarly, to the genus *Diutina*, barcoding gap analysis of the *Pichia* clade revealed barcoding gaps for the primary barcode, secondary barcode and the combination of both barcodes (Figure 4), resulting in an accurate identification of all species of the *Pichia* clade.

#### *Scedosporium* Species

Fungi belonging to the genus *Scedosporium* cause a wide variety of diseases from localized infections to invasive diseases (Cortez et al., 2008). *Scedosporium* species mainly cause infections in patients with a compromised immune systems largely due to underlying illnesses such as solid organ transplantation, cystic fibrosis, leukemia and bone marrow transplantation (Tamm et al., 2001; Allen et al., 2013; Kubisiak-Rzepczyk et al., 2013; Yu et al., 2013; Johnson et al., 2014). *Scedosporium* spp. can also cause infection in immunocompetent patients (Ceccarelli et al., 2012; Agatha et al., 2014; Cruysmans et al., 2015).

The taxonomy of the genus *Scedosporium* is complex and has changed multiple times over the last decade. Previously, the nomenclature of the genus ruled *Scedosporium* to be the anamorph name whilst *Pseudallescheria* and *Petriella* were the teleomorph names (Rainer and de Hoog, 2006; Cortez et al., 2008; Lu et al., 2011). The two major clinical species were *Scedosporium prolificans* and *Scedosporium apiospermum* and its teleomorph form, *Pseudallescheria boydii* (Cortez et al., 2008). Multilocus sequencing, morphological analysis and physiological testing later demonstrated that *S. apiospermum* and *P. boydii* are separate species and not anamorphs teleomorph pairs (Gilgado et al., 2008). In 2011, the new nomenclature rules dictating “one fugus = one name” led to the fact that *Scedosporium* took precedence over *Pseudallescheria* as the genus name (Hawksworth, 2011; Lackner et al., 2014). The composition of the genus was also changed with *S. prolificans* being removed from the genus and renamed to *Lomentospora prolificans* (Lackner et al., 2014). The separation between *S. apiospermum* and *S. boydii* was reinforced, however, it was noted that there was no clinical difference between the species, as such all species are referred to the *S. apiospermum* species complex (Lackner et al., 2014).

In this study, there was no barcoding gap present for the primary barcode indicating that *Scedosporium* species cannot be accurately identified using this region alone (Figure 4). The predominant reason for the overlap in intraspecies and interspecies genetic distances was the high similarity between *S. apiospermum* and *S. boydii*. This was reflective of the nomenclature history as these species were previously thought to be an anamorph – teleomorph pair. Barcoding gap analysis of the secondary barcoding region introduced a barcoding gap and hence allowed for the accurate identification of all *Scedosporium* species (Figure 4). As the separation of *S. apiospermum* and *S. boydii* was established via multilocus sequence analysis, it is reassuring that *TEF1α* can resolve all *Scedosporium* species to the same degree (Gilgado et al., 2008). With the introduction of the dual barcoding scheme, all *Scedosporium* species can now be accurately identified.

### Assessment of the Dual Barcoding Scheme

Meaningful implementation of the dual DNA barcoding scheme depends on demonstration of an improvement in the identification accuracy of fungal species causing infections when adding the secondary barcode, *TEF1α*. To demonstrate this the two fungal barcodes were analyzed separately and in combination from selected species, which had sequences from both barcodes being in the “ISHAM Barcoding Database,” using barcoding gap analysis through the generation of genetic distances by the K2P model (Kimura, 1980).

Although K2P has been used in all DNA barcoding studies since its introduction by Hebert et al. (2003), the accuracy of this model has been questioned (Kimura, 1980; Hebert et al., 2003). In the selection of ITS region as the primary fungal DNA barcode, the uncorrected p-distances were used for barcoding gap analysis whereas in the case of the secondary fungal DNA barcode (*TEF1α*) analysis the K2P method was applied (Schoch et al., 2012; Stielow et al., 2015). The K2P region was selected to generate barcoding gaps over uncorrected p-distances as K2P has been found to generate larger barcoding gaps for most data sets (Srivathsan and Meier, 2012). Additionally, in datasets where uncorrected p-distances were preferred, the performance of K2P was found to be similar (Srivathsan and Meier, 2012). More complicated models have been applied in some studies in an attempt to improve the accuracy of the K2P model, however, the differences were minimal, prompting us to use K2P in our study (Collins et al., 2012; Barley and Thomson, 2016).

Comparison of the number of pathogenic fungal species and strains represented in the “ISHAM Barcoding Database” highlighted the deficit in the number of *TEF1α* reference sequences. Therefore, the value of this study is limited as only strains with both barcodes available were used for the analysis. More species represented by more sequences would give a more exact assessment about the value and resolution power of the dual barcoding concept.

DNA barcode gab analysis showed that two of the groups (*Diutina* and *Pichia*) tested were accurately identified using the primary barcode and the secondary barcode on its own, demonstrating that both barcodes had similar resolution. For the remaining two groups (*Lodderomyces* and *Scedosporium*) the primary barcode was not able to produce barcoding gaps whilst the secondary did, demonstrating that the secondary barcode had a higher resolution power. In addition, the combination of both barcodes increased the discriminatory power enabling a more accurate identification. These results indicate, that the application of the dual barcoding system drastically improves species identification in cases where a single barcoding system is unable to do so.

### Implementation of the Dual Barcoding Scheme

**FIGURE 5:**
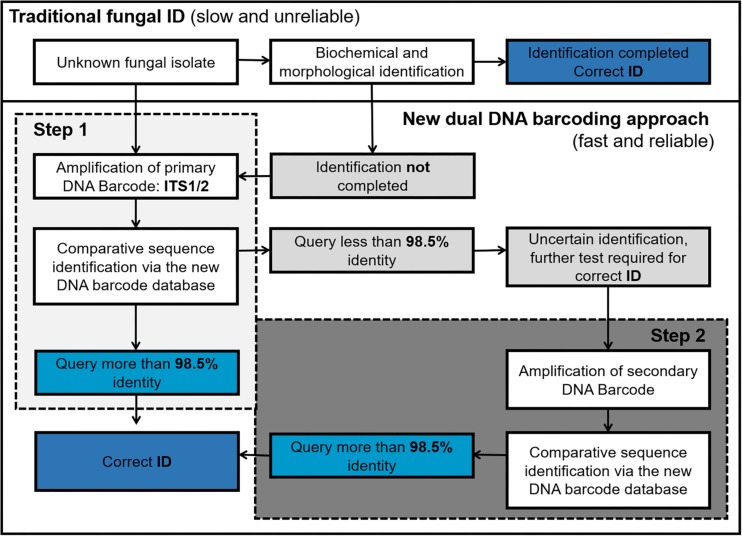
Workflow of DNA barcoding based fungal ID (Steps 1 and 2 can be done in parallel to speed up the process).

We envisage that the proposed dual barcoding scheme can be applied in the routine diagnostic setting in a stepwise procedure. After a clinical specimen is obtained, the unknown fungal isolate is first assessed based on its morphologic and/or biochemical characteristics. Then those unknown fungal isolates which lack obvious morphological characteristics or result in unclear biochemical profiles should be subjected to DNA isolation and primary fungal DNA barcoding (ITS1/2 region). If the obtained sequence shows less than 98.5% identity to a given ITS reference sequence in the database, the secondary fungal DNA barcode (*TEF1α*) should be obtained to achieve a final species identification (Figure 5).

The dual fungal DNA barcoding scheme is an efficient diagnostic tool for the identification of agents of invasive mycoses and can be confidently implemented into routine diagnostics. Its implementation along with the expansion of the “ISHAM Barcoding Database” will lead to a paradigm shift in fungal disease diagnostics, enabling a highly accurate identification of the agents of fungal disease. The timely initiation of appropriate therapy will improve patient outcomes through the reduction in morbidity and mortality, prevent the use of unnecessary or inappropriate antifungal drugs, minimizing drug toxicity and resistance development and greatly reduce the associated health care cost.

## Call for Data Submission

To achieve a 100% coverage of all pathogenic fungi and reflect the intraspecies variation for both the primary and secondary fungal DNA barcode we call for the submission of quality-controlled reference sequences of the ITS region and the *TEF1α* for inclusion into the “ISHAM Barcode Database.” Please contact the curators of the database WM at wieland.meyer@sydney.edu.au and LI at laszlo.irinyi@sydney.edu.au.

## Data Availability

The primary and secondary fungal barcodes generated and analyzed for this study are available in the ISHAM Barcoding Database (http://its.mycologylab.org/).

## Author Contributions

WM, SC, and TS conceived the study. WM coordinated the study and supervised the molecular and data analysis. All members of the ISHAM working group for “Barcoding of Medical Fungi” performed the sample collections. MH and LI conducted the sample processing, all molecular studies, and data analysis. MH, LI, and WM performed the data interpretation. All authors wrote and corrected the manuscript.

## Conflict of Interest Statement

The authors declare that the research was conducted in the absence of any commercial or financial relationships that could be construed as a potential conflict of interest.

## Footnotes

1 http://its.mycologylab.org/
