# Supplementary material for: Dual DNA Barcoding for the Molecular Identification of the Agents of Invasive Fungal Infections
Source: Front Microbiol. 2019 Jul 18;10:1647. doi: 10.3389/fmicb.2019.01647 (PMC6657352; doi:10.3389/fmicb.2019.01647)
Supplement: Supplementary file 1 [file Table_1.DOCX]

Supplementary Material

## Supplementary Tables

**Supplementary Table 1.** Fungal species and number of strains for which new *translational elongation factor 1α* (*TEF1α*) sequences have been generated as part of this project

| **Species** | **Number of Strains** |
| --- | --- |
| *Aspergillus amstelodami* | 1 |
| *Aspergillus flavus* | 3 |
| *Aspergillus fumigatus* | 1 |
| *Aspergillus niger* | 2 |
| *Aspergillus tubingensis* | 1 |
| *Blastobotrys adeninivorans* | 1 |
| *Blastobotrys proliferans* | 1 |
| *Candida albicans* | 5 |
| *Candida blankii* | 1 |
| *Candida boleticola* | 1 |
| *Candida caryicola* | 1 |
| *Candida chilensis* | 1 |
| *Candida dubliniensis* | 6 |
| *Candida duobushaemulonii* | 1 |
| *Candida entomophila* | 1 |
| *Candida fructus* | 2 |
| *Candida glabrata* | 2 |
| *Candida glaebosa* | 1 |
| *Candida haemuloni* | 1 |
| *Candida hawaiiana* | 1 |
| *Candida incommunis* | 2 |
| *Candida inconspicua* | 8 |
| *Candida insectalens* | 1 |
| *Candida insectorum* | 1 |
| *Candida intermedia* | 2 |
| *Candida membranifaciens* | 1 |
| *Candida mesenterica* | 1 |
| *Candida metapsilosis* | 1 |
| *Candida multi-gemmis* | 1 |
| *Candida orthopsilosis* | 1 |
| *Candida parapsilosis* | 16 |
| *Candida railenensis* | 1 |
| *Candida santamariae* | 2 |
| *Candida savonica* | 2 |
| *Candida sequanensis* | 1 |
| *Candida sophiae-reginae* | 1 |
| *Candida tropicalis* | 4 |
| *Candida zeylanoides* | 1 |
| *Cladosporium sphaerospermum* | 1 |
| *Clavispora lusitaniae* | 4 |
| *Cryptococcus gattii* | 44 |
| *Cryptococcus magnus var. magnus* | 3 |
| *Cryptococcus neoformans* | 9 |
| *Cutaneotrichosporon curvatum* | 1 |
| *Danielozyma litseae* | 1 |
| *Danielozyma ontarioensis* | 1 |
| *Dekkera bruxellensis* | 1 |
| *Diutina catenulata* | 1 |
| *Epidermophyton floccosum* | 1 |
| *Fusarium proliferatum* | 2 |
| *Galactomyces geotrichum* | 3 |
| *Geotrichum candidum* | 3 |
| *Geotrichum capitatum* | 1 |
| *Groenewaldozyma salmanticensis* | 1 |
| *Hannaella luteola* | 1 |
| *Hyphopichia homilentoma* | 1 |
| *Hyphopichia rhagii* | 1 |
| *Kluyveromyces lactis* | 1 |
| *Kluyveromyces marxianus* | 10 |
| *Kodamaea ohmeri* | 5 |
| *Kuraishia capsulata* | 1 |
| *Magnusiomyces capitatus* | 4 |
| *Meyerozyma guilliermondii* | 5 |
| *Millerozyma farinosa* | 2 |
| *Naganishia albida* | 3 |
| *Nakazawaea ernobii* | 2 |
| *Nakazawaea holstii* | 1 |
| *Papiliotrema aurea* | 1 |
| *Penicillium brevicompactum* | 1 |
| *Penicillium chrysogenum* | 1 |
| *Pichia kudriavzevii* | 7 |
| *Pichia membranifaciens* | 4 |
| *Scedosporium aurantiacum* | 4 |
| *Scedosporium boydii* | 1 |
| *Scheffersomyces coipomensis* | 1 |
| *Sporobolomyces lactosus* | 1 |
| *Starmella apicola* | 1 |
| *Suhomyces canberraensis* | 1 |
| *Suhomyces prunicola* | 1 |
| *Suhomyces tanzawaensis* | 1 |
| *Torulaspora delbrueckii* | 2 |
| *Trichomonascus ciferrii* | 3 |
| *Trichosporon asahii* | 2 |
| *Vishniacozyma carnescens* | 5 |
| *Wickerhamomyces anomalus* | 13 |
| *Yamadazyma mexicana* | 3 |
| *Yamadazyma scolyti* | 3 |
| *Yarrowia galli* | 1 |
| *Yarrowia lipolytica* | 17 |
| *Zygoascus hellenicus* | 1 |
